# Prognostic Value Analysis of Mutational and Clinicopathological Factors in Non-Small Cell Lung Cancer

**DOI:** 10.1371/journal.pone.0107276

**Published:** 2014-09-08

**Authors:** Chenguang Li, Ligang Hao, Yue Li, Shengguang Wang, Hui Chen, Lianmin Zhang, Bin Ke, Yuesong Yin, Haijin Suo, Bingsheng Sun, Bin Zhang, Changli Wang

**Affiliations:** 1 Department of Lung Cancer, Tianjin Medical University Cancer Institute and Hospital, National Clinical Research Center for Cancer, Tianjin, China; 2 Key Laboratory of Cancer Prevention and Therapy, Tianjin, China; 3 Tianjin Lung Cancer Center, Tianjin, China; 4 Department of Gastric Cancer, Tianjin Medical University Cancer Institute and Hospital, Tianjin, China; 5 Department of Surgery, Tianjin Beichen Hospital, Tianjin, China; H. Lee Moffitt Cancer Center & Research Institute, United States of America

## Abstract

**Introduction:**

Targeting activating oncogenic driver mutations in lung adenocarcinoma has led to prolonged survival in patients harboring these specific genetic alterations. The prognostic value of these mutations has not yet been elucidated. The prevalence of recently uncovered non-coding somatic mutation in promoter region of *TERT* gene is also to be validated in lung cancer. The purpose of this study is to show the prevalence, association with clinicalpathological features and prognostic value of these factors.

**Methods:**

In a cohort of patients with non-small cell lung cancer (NSCLC) (n = 174, including 107 lung adenocarcinoma and 67 lung squamous cell carcinoma), *EGFR*, *KRAS*, *HER2* and *BRAF* were directly sequenced in lung adeoncarcinoma, ALK fusions were screened using FISH (Fluorescence *in situ* Hybridization).*TERT* promoter region was sequenced in all of the 174 NSCLC samples. Associations of these somatic mutations and clinicopathological features, as well as prognostic factors were evaluated.

**Results:**

*EGFR*, *KRAS*, *HER2*, *BRAF* mutation and *ALK* fusion were mutated in 25.2%, 6.5%, 1.9%, 0.9% and 3.7% of lung adenocarcinomas. No *TERT* promoter mutation was validated by reverse-sided sequencing. Lung adenocarcinoma with *EGFR* and *KRAS* mutations showed no significant difference in Disease-free Survival (DFS) and Overall Survival (OS). Cox Multi-variate analysis revealed that only N stage and *HER2* mutation were independent predictors of worse overall survival (HR = 1.653, 95% CI 1.219–2.241, *P* = 0.001; HR = 12.344, 95% CI 2.615–58.275, *P* = 0.002).

**Conclusions:**

We have further confirmed that *TERT* promoter mutation may only exist in a very small fraction of NSCLCs. These results indicate that dividing lung adenocarcinoma into molecular subtypes according to oncogenic driver mutations doesn't predict survival difference of the disease.

## Introduction

Lung cancer is one of the most devastating diseases and the leading cause of cancer-related deaths worldwide [1]. Non-small cell lung cancer (NSCLC), which accounts for about 85% of lung cancer cases, is further divided to histological subtypes of adenocarcinoma (ADC), squamous cell carcinoma (SCC) and large cell carcinoma. Adenocarcinoma has become the most common lung cancer subtype [2]. The 5-year survival rate of NSCLC remains low as 15–16%, despite improvement of treatment in the past decades [1], [3]. Better understanding of the NSCLC tumorigenesis and factors associated with prognosis is needed.

Uncovering of activating mutations in tyrosine kinase domain of *EGFR* has led to developing and wide use of gefetinib and erlotinib, which has proven to be effective in the treatment for part of *EGFR* mutated lung adenocarcinomas [2], [4]. According to specific oncogenic driver mutations, lung adenocarcinoma is divided to molecular subtypes such as *EGFR*, *KRAS*, *ALK*, *HER2*, *BRAF* and so on [5], [6]. Most of these driver genes have specific targeted drugs in use or clinical trials. Compared to adenocarcinoma, knowledge on genetic alterations of lung squamous cell carcinoma is limited. Frequent mutation, amplification or loss of *FGFR1*, *PTEN*, *PIK3CA*, *NFE2L2*, *NRF* have been identified in comprehensive genomic study in lung squamous cell carcinoma [7]. Because the largely undefined mutational spectrum and low frequencies of these genes, targeted therapies in lung squamous cell carcinoma is lagging behind and constricted to clinical trials.

Recently, human cancer genome sequencing studies have revealed somatic mutaitons in the core promoter of human telomerase reverse transcriptase (*hTERT*).

Gene, which codes the catalytic subunit of telomerase [8]–[10]. Either C228T or C250T of *hTERT* promoter mutations elevated transcriptional activity of *hTERT* gene in the luciferase reporter assays [8]. The tumor types with frequent *hTERT* promoter mutations included Melanoma (71%), Glioma (51%), Myxoid liposarcoma (79%), Urothelial carcinoma of bladder (66%) [8], [9]. Evidences in above studies suggested that *hTERT* promoter mutations potentially act as a driver gene in melanoma. However, the prevalence of *hTERT* promoter mutation in lung cancer clinical samples is absent in these studies. It's necessary to show the prevalence of *hTERT* promoter mutation in NSCLC samples as lung cancer shares driver mutated genes with melanoma, such as *BRAF* and *NRAS* [8], [9], [11].

In this study, we aimed to show the comprehensive molecular and clinicopathological features in NSCLC, as well as the prognostic value of these characteristics.

## Methods

### Patients and tissues

Primary tumor samples were obtained from 174 patients who underwent potentially curative pulmonary resection at the Tianjin Medical University Cancer Hospital from Jan 2004 through Jan 2008. This study was approved by the Institutional Review Board of Tianjin Medical University Cancer Institute and Hospital. All participants gave written informed consent. Patients were enrolled in this specific study based upon the following criteria: they had a pathologic diagnosis of lung adenocarcinoma or squamous cell carcinoma, their tumor sample contained a minimum of 50% tumor cells as determined by study pathologists, they did not receive neoadjuvant chemotherapy, and they had sufficient tissue for molecular analysis. Clinicopathological features and prognosis information were collected.

### DNA isolation and mutational analysis

Genomic DNA were isolated from Formalin-Fixed Paraffin-Embedded (FFPE) samples according to standard protocols (TIANquick FFPE DNA Kit, Tiangen Inc, China). *EGFR* (exons 18–22), *HER2* (exon 20), *KRAS* (exons 2 to 3), *BRAF* (exon 15) and *TERT* promoter region were PCR amplified using DNA and directly sequenced. Multiplex PCR analysis was done with rTaq DNA polymerase (Toyobo, Osaka, Japan). Primers used in these experiments are listed in **Table S1**. ALK fusions were screened using FISH with FFPE slides.

### Statistical analysis

Associations between mutations and clinical and biological characteristics were analyzed by *χ^2^* or Fisher's exact test. Survival curves were drawn by the Kaplan–Meier method. The Cox proportional hazards regression (forward likelihood ratio model) was used for multivariate survival analyses. All data were analyzed using the Statistical Package for the Social Sciences Version 16.0 Software (SPSS Inc., Chicago, IL). The two-sided significance level was set at *P*<0.05.

## Results

### Patient Characteristics

One hundred and seven lung ADC and 67 SCC patients were enrolled in this study, including 105 men and 69 women (average 58 years; range, 39–77 years). There were 109 smokers and 65 non-smokers, accounting for 63% and 37%, respectively. The number of patients in stages I-III was 57, 46, and 69, respectively. Two samples were with undefined stage. The detailed information is listed in **Table 1**.

**Table 1:** Demographics and clinicopathologic features of 174 patients with NSCLC.

Variables	No. of patients (%)
Age	
≤50	37(21.3)
51–60	69(39.6)
61–70	49(28.2)
>70	19(10.9)
Mean ± SD (range)	58.17±9.44
Histologic subtype	
Adenocarcinoma	107(61.5)
Squamous cell carcinoma	67(38.5)
Smoking status	
Smoker	109(62.6)
Non-smoker	65(37.4)
Stage	
IA	24(13.8)
IB	33(18.9)
IIA	30(17.2)
IIB	16(9.2)
IIIA	68(39.1)
IIIb	1(0.5)
NA	2(1.1)
Total	174

SD,standard deviation; NA,not applicable.

### *EGFR, KRAS, HER2* and *BRAF* Mutation Status and ALK fusions

**Figure 1:**
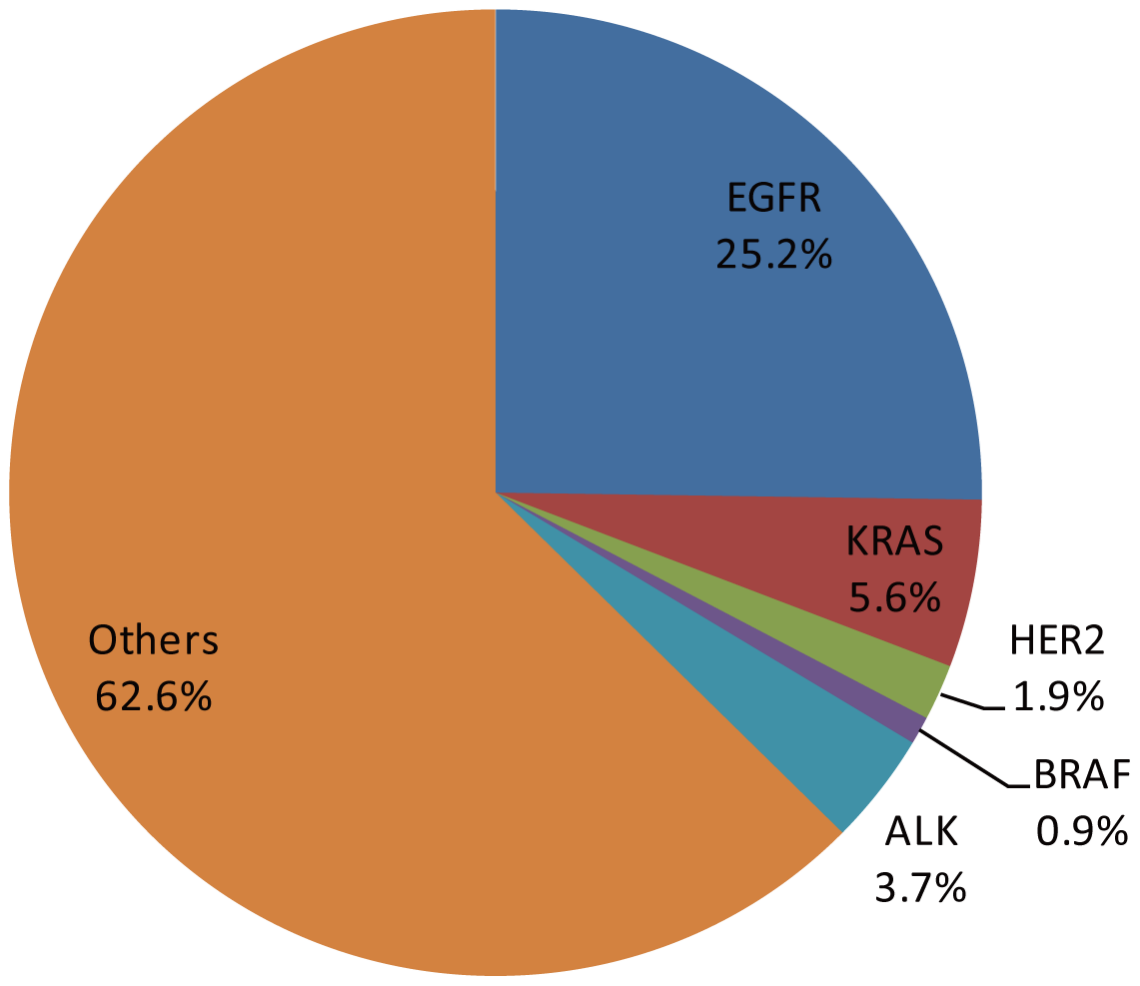
Pie chart of *EGFR, KRAS, HER2* and *BRAF* mutation spectrum from 107 lung adenocarcinomas. The mutation rates of *EGFR, KRAS, HER2* and *BRAF* were 25.2, 5.6, 1.8 and 0.9%, respectively.

Of the 107 lung adenocarcinoma samples, 25.2% (27/107) of tumors were found to harbor *EGFR* kinase domain mutations. Among these, 17 were deletions in exon 19 and 6 were L858R missense changes. Other alterations included 1 exon 20 insertion, exon 20 D807N and T790A mutations, 1 exom 18 R705K mutation. Six samples (5.6%) had a *KRAS* mutation, including five G12V mutations and an I36M missense mutation. Two samples harbored *HER2* exon 20 mutation, including a 776 to 779 YVMA insertion and a S789P mutation. Only one *BRAF* L588F missense mutation was identified in these tumor samples (**Figure 1**). ALK fusions were detected in 4 (3.7%) samples. Because mutations of *EGFR, KRAS, HER2, BRAF*, as well as *ALK* fusions have been well established to be existed in lung adenocarcinoma [5], [12]–[14], these mutation and fusion genes that ‘drives’ lung adenocarcinoma were not screened in lung squamous cell carcinoma.

### 5′ non-coding region mutations of *TERT* gene in NSCLC

Neither C228T nor C250T mutations was validated in the 174 NSCLC samples after reverse sequencing. These results indicated that *TERT* promoter mutation may only exist in a very small fraction of primary NSCLC samples.

### Survival Analysis

**Figure 2:**
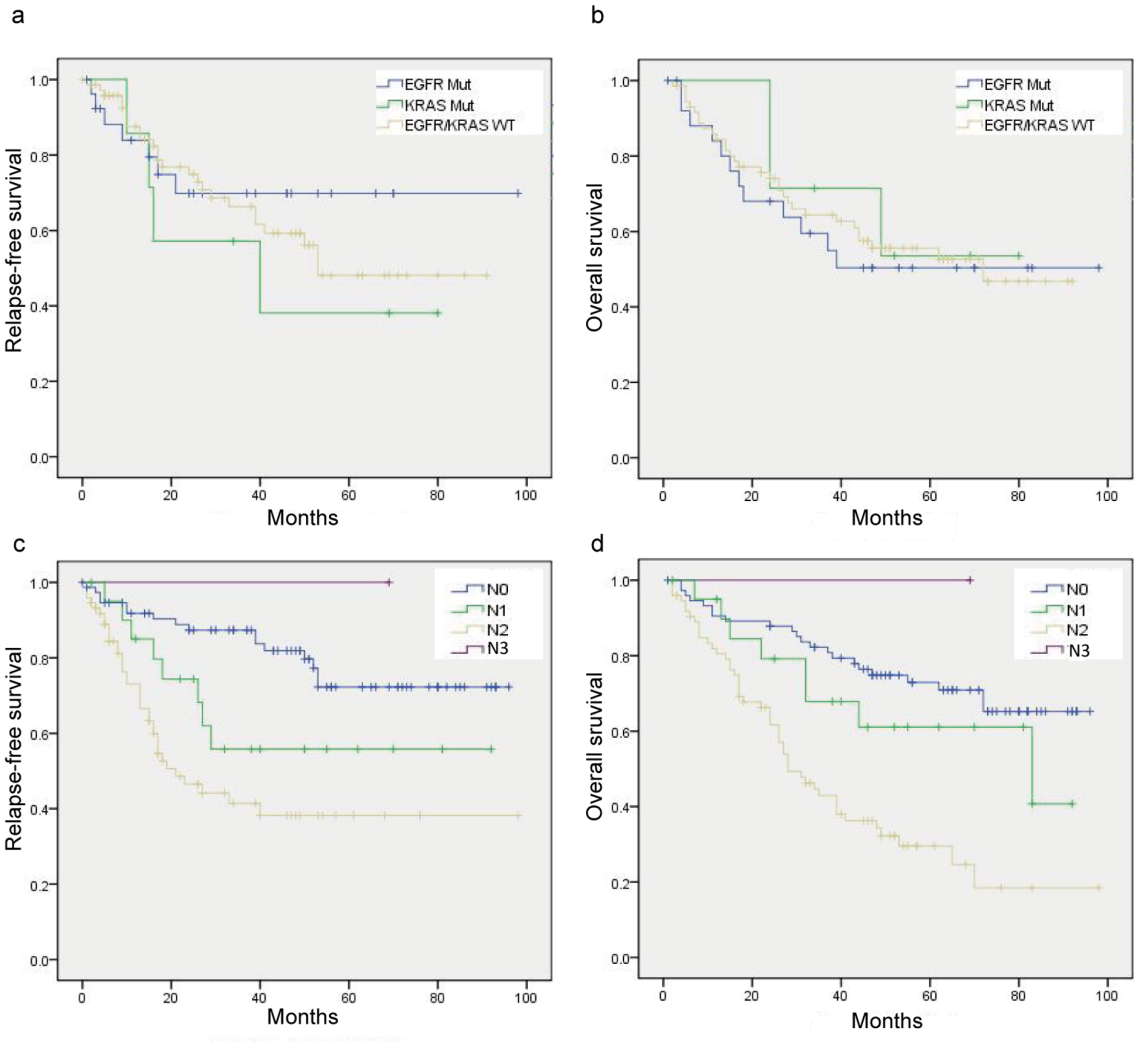
Relapse-free survival and overall survival in patients with NSCLC. *EGFR* and *KRAS* mutation status were not associated with relapse-free survival (*P* = 0.600) or overall survival (*P* = 0.873) in lung adenocarcinoma; **c–d**. Lymphnode stage (N stage) was significantly associated with relapse-free survival (*P* = 0.001) or overall survival (*P* = 0.001) in NSCLC.

By univariate analysis, advanced clinical staging (*P* = 0.018), lymphnode metastasis (*P*<0.001), ≥2 station of metastatic lymphnode (*P*<0.001), received adjuvant chemotherapy (*P* = 0.004) were associated disease relapse (**Table 2**). *EGFR* and *KRAS* mutation status were not associated with relapse-free survival (*P* = 0.600) or overall survival in lung adenocarcinoma (*P* = 0.873) (**Figure 2**). Cox multivariate forward stepwise analysis (adjusting for age, gender, smoking history, clinical stage, histologic subtypes and mutational status) revealed that only lymphnode stage (N stage) and *HER2* mutation was independent predictors of poorer overall survival (HR 1.653, 95% CI 1.219–2.241; *P* = 0.001; HR 12.344, 95% CI 2.615–58.275; *P* = 0.002). Detailed mutational and survival information is listed in **Table S2**.

**Table 2:** Association between clinical and molecular factors and disease relapse.

Factors	Relapse		*P*
Clinical Stage	Relapsed	Relapse-free	
I	13	46	0.018
II	16	30	
III	32	37	
EGFR status			
Mutated	7	20	0.326
Wildtype	29	51	
KRAS status			
Mutated	4	3	0.188
Wildtype	32	68	
Lymphnode metastasis			
Positive	44	52	0.001
Negative	17	61	
No.of metastatic lymphnode station			
<2	29	83	0.001
≥2	32	30	
Adjuvent Chemotherapy			
Received	45	58	0.004
Not-received	16	55	

## Discussion

NSCLC is a heterogeneous disease, outcome differs even in patients with identical clinicopathologic features. Identification of oncogenic driver mutations in lung adenocarcinoma has greatly promoted clinical use and development of targeted drugs [5], [6], [15], [16]. Though *EGFR* and *KRAS* mutations have been established as predictive and prognostic markers for patients who receive *EGFR*-TKIs [4], [17], [18], results on the prognostic value of these alterations at genomic level in patients who didn't receive *EGFR*-TKIs were rarely reported.

We have screened the prevalence of known driver mutations of *EGFR*, *KRAS*, *HER2, BRAF* and *ALK* in a cohort of 107 lung adenocarcinoma sample with complete prognostic information. The *EGFR* mutation, *KRAS* mutation and *EGFR*/*KRAS* wild type groups showed no significant difference in both relapse-free survival and overall survival in the present study. All the patients enrolled in this study didn't receive small molecular drugs targeting *EGFR*. Our results suggest that specific driver mutation of *EGFR* or *KRAS* gene doesn't predict survival advantage or disadvantage when these patients didn't receive specific target therapies. In the multivariate analysis, *HER2* mutation was identified as independent predictor of unfavorable prognosis. Because only two samples harbored *HER2* mutation, further study with larger number of samples are needed to confirm this result. Based on these facts, we conclude that to divide lung adenocarcinoma into molecular subtypes only predicts therapy response but not survival benefit in patients who didn't receive these drugs.

The emerging of somatic mutations of *hTERT* gene 5′ promoter region in melanoma has provided another promising candidate driver gene for lung cancer. Several studies have identified frequent *hTERT* 5′ promoter mutations in melanoma [8], [10], gliomas, as well as from relatively low rates of self renewal tissues like liposarcomas, hepatocellular carcinomas, urothelial carcinomas, squamous cell carcinomas of the tongue and medulloblastomas [9], [19]. We screened the *hTERT* 5′ promoter region in 174 NSCLC FFPE samples to explore the mutation rate and potential prognostic value. We identified only three C250T mutations from the 174 samples, but none of these were validated by reverse sequencing, which could be possibly caused by DNA damage in the process of formalin fixing and paraffin embedding. These results confirmed that *hTERT* 5′ promoter mutations may only exist in a very small fraction of NSCLC cases.

The limitation of the present study should be discussed. In order to explore the prognostic significance, we used FFPE samples which have complete survival information. However, the sensitivity of sequencing results is affected by the relatively low quality of FFPE samples, in which DNA is degraded and artificially mutated. The mutation rate of well known driver genes is lower than previous studies using high quality snap-frozen samples [20], [21], and the artificial mutations appeared in *hTERT* 5′ promoter region. Though consecutive samples with complete survival data are not always available, our study provided the mutation spectrum and prognostic association of these genetic alterations with available sample and techniques.

In conclusion, our work illustrated the mutation spectrum of *EGFR*, *KRAS*, *HER2, BRAF* and *ALK* in lung adenocarcinoma, as well as confirmed that h*TERT* 5′ promoter region mutation rarely occurred in NSCLC samples. We also confirmed that these driver mutations lack prognostic value in lung adenocarcinoma patients who didn't receive EGFR TKI treatment, and subtypes divided by these oncogenic driver mutations don't predict significant survival difference.

## Supporting Information

Table S1 List of primers used for polymerase chain reaction amplification of the EGFR, KRAS, HER2 and BRAF gene.
(DOCX)

Table S2 Mutational and survival information of 174 NSCLC patients. Sex, 1 male, 2 female; Smoking, 0 non-smoker, 1 smoker; path, pathology; WT, wild-type; DEL, deletion; INS, insertion; NEG, negative; POS, positive.
(XLS)
