# Supplementary material for: Prognostic Value Analysis of Mutational and Clinicopathological Factors in Non-Small Cell Lung Cancer
Source: PLoS One. 2014 Sep 8;9(9):e107276. doi: 10.1371/journal.pone.0107276 (PMC4157862; doi:10.1371/journal.pone.0107276)
Supplement: Table S1 — List of primers used for polymerase chain reaction amplification of the EGFR, KRAS, HER2 and BRAF gene. (DOCX) [file pone.0107276.s001.docx]

| **Supplementary Table 1**. List of primers used for polymerase chain reaction amplification of the EGFR, KRAS, HER2 and BRAF gene | | |
| --- | --- | --- |
| Exons | Forward primer (5'→3') | Reverse primer (5'→3') |
| EGFR E18 | FCCTTGTCTCTGTGTTCTTGT | CTGCGGCCCAGCCCAGAGGC |
| EGFR E19 | CATGTGGCACCATCTCACA | CCACACAGCAAAGCAGAAAC |
| EGFR E20 | CCATGCGAAGCCACACTGA | CGTATCTCCCTTCCCTGATTACC |
| EGFR E21 | CAGGGTCTTCTCTGTTTCAG | TAAAGCCACCTCCTTACTTT |
| KRAS E2 | GTGTGACATGTTCTAATATAGTCA | GAATGGTCCTGCACCAGTAA |
| HER2 E20 | GGGTGTGTGGTCTCCCATAC | GCAAAGAGCCCAGGTGCATA |
| BRAF E15 | TCATAATGCTTGCTCTGATAGGA | GGCCAAAAATTTAATCAGTGGA |
